# Addition of Chickpea Flour in Durum Wheat Flour Makes Tortilla More Nutritious and Palatable, and Technologically Acceptable

**DOI:** 10.3390/foods12010072

**Published:** 2022-12-23

**Authors:** Asmaa Benayad, Mona Taghouti, Aouatif Benali, Abdelmajid Zouahri, Samir Bikri, Youssef Aboussaleh, Nadia Benbrahim, Shiv Kumar

**Affiliations:** 1 Department of Life Sciences, Faculty of Sciences, University Ibn Tofail (UIT), Kenitra 14000, Morocco; 2 Research Unit of Plant Genetic Resources and Plant Breeding, National Institute for Agronomic Research (INRA), Rabat 10101, Morocco; 3 Research Laboratory of Food Technology, National Institute for Agronomic Research (INRA), Rabat 10101, Morocco; 4 Research Unit of Environment and Conservation of Natural Resources, National Institute for Agronomic Research (INRA), Rabat 10101, Morocco; 5 Biodiversity and Crop Improvement Program, International Center for Agricultural Research in the Dry Areas (ICARDA), Rabat 10101, Morocco

**Keywords:** durum wheat, chickpea, flours, blends, tortilla, quality, Morocco

## Abstract

In order to contribute to the reduction of nutritional deficiencies in Morocco, this study was undertaken to develop a healthier tortilla with higher iron and protein, while maintaining adequate technological and sensory qualities. Composite durum wheat flour enriched with 20, 25, 30, and 35% chickpea flour was assessed for nutritional, functional, and technological properties. Then, we selected two composite blends of 75:25 and 70:30 of durum wheat and chickpea flours for making tortillas to study nutritional, technological, and sensorial qualities. In addition, we studied the effects of making and cooking process and storage time. Kruskal–Wallis and Mann—Whitney tests were used for data analysis, and GraphPad Prism was used to create graphs. The results showed that composite tortilla had significantly higher nutritional value than durum wheat tortilla, and the best ratio was 30% chickpea flour. At this ratio, the results showed the best cooking time and the best yellowness, but tortilla fluffiness and puffiness decreased. Tortilla processing significantly increased protein at 30% chickpea flour, while minerals except sodium, weight, and diameter decreased. Adding 30% chickpea flour to durum wheat tortilla improved flavor. Then, storage decreased the weight resulting in decreased flexibility, and sanitary quality was lost early for 30% chickpea flour. In conclusion, adding 30% chickpea flour to durum wheat flour results in a healthier and tastier tortilla, which should be consumed fresh.

## 1. Introduction

Durum wheat (*Triticum durum* Desf.) is one of the most important food crops in the Mediterranean region because of its various end-products such as bread, pasta, macaroni, and couscous. In Morocco, durum wheat is one of the oldest cultivated cereals [1] which plays an important role in its food security. Nevertheless, nutritional status of Moroccans is characterized by malnutrition [2]. Urbanization, economic development, and globalization are at the origin of changes in food habits [3]. Indeed, considering diet evolution towards fast foods such as burritos, tacos, tortillas, pasta, macaroni, etc., which are rich in energy but lack in sufficiently balanced essential minerals and proteins, the present fast food-based diets have led to triple burden of malnutrition including overweight and obesity. In Morocco, iron deficiency is the most widespread mineral deficiency [4] causing anemia, which has been recognized as a serious health issue [5]. Thus, development of nutritious and safe food products is one of the major tasks to prevent the so-called lifestyle diseases. Diversified food is a prerequisite to meet nutritional requirements of a human body. Food fortification with different combinations of food ingredients has traditionally been in practice to decrease nutrient deficiencies [6]. In Morocco, numerous studies [7,8,9] have considered cereal-pulse mixtures as an approach to mitigate the impact of malnutrition. Chickpea (*Cicer arietinum* L.) is nutritionally rich in terms of protein, dietary fibers, essential minerals, and vitamins [10]. Incorporating chickpea flour into durum wheat flour to improve nutritional value of flat bread is an age-old practice worldwide particularly in the Mediterranean region, the Middle East, China, Indian subcontinent, and Central America [11]. However, no efforts were made to study the right combination of the blends and their nutritional values. Further, no study was undertaken on nutritional quality of tortilla made from durum wheat flour fortified with chickpea flour. In this context, the present study was undertaken to evaluate nutritional, functional, and technological properties of durum wheat-chickpea composite flours using different combinations and of tortilla made from such blends. The aim was to ensure improved nutritional quality of tortilla while preserving adequate technological and sensory qualities.

## 2. Material and methods

### 2.1. Raw Material

Durum wheat variety “Louiza” and chickpea (Kabuli) variety “Farihane”, listed in the official national catalog, were milled into whole meal flour using UDY Cyclone and mini–Hammer mill equipped with a 1 mm and 0.5 sieves respectively. Durum wheat flour (control) was substituted for four combinations of 20, 25, 30, and 35% of chickpea flour. Nutritional, functional, and technological properties were evaluated, in triplicate, for each composite compared to the checks, durum wheat and chickpea flours. Then, nutritional, technological, and sensory qualities were assessed for tortilla made from such durum wheat-chickpea blends.

### 2.2. Nutritional Attributes

Flours and composite flours were analyzed for quality traits including physico-chemical composition (moisture, crude ash, crude proteins, crude fibers, and crude fat) following American Association of Cereal Chemists (AACC) approved methods [12]. Total carbohydrates were estimated by difference, by subtracting moisture, crude ash, crude proteins, and crude fat values from 100% [13], and energy value was calculated using the Atwater conversion factors, where energy value = [9 × crude fat (%) + 4 × crude proteins (%) + 4 × total carbohydrates (%)] [14]. Mineral content was determined on dry matter (dm) basis through extraction from the samples using dry ashing method [15]. Iron, zinc, copper, calcium, and magnesium were measured by atomic absorption spectrophotometry (SpectrAA 220FS, Varian Inc., Palo Alto, CA, USA) [16]. Sodium and potassium contents were estimated using flame photometry (BWB XP, BWB Technologies Inc., Berkshire, UK) [13], and phosphorus was measured by ammonium molybdate method [14] using spectrophotometric methods [17]. Total phenolic compounds were measured spectrophotometrically (GENESYS 10S UV–Vis, Thermo Scientific Inc., Waitham, MA, USA) according to the Folin–Ciocalteu method, using gallic acid as a standard [18] and expressed as mg gallic acid equivalents/g extract (mg GAE/g extract). Condensed tannins were measured using the modified vanillin–HCl in methanol method [19] and expressed as mg catechin equivalents/g extract (mg CE/g extract). Total flavonoids were measured according to [18] and expressed as mg quercetin equivalents/g extract (mg QE/g extract), and anti-radical activity was measured using the widely accepted method: DPPH radical-scavenging activity [20].

### 2.3. Functional Properties

Bulk density was determined using [21] method. About 10 g sample was weighed in a 50 mL graduated cylinder which was gently tapped 10 times on a laboratory bench from a height of 5 cm. Then, sample volume was recorded and the bulk density was calculated using the Equation (1):

(1) $$\mathrm{BD}\left(\mathrm{mL}\right)=\left(\frac{a}{b}\right)$$

where BD = bulk density, a = sample weight, and b = tapped sample volume.

Swelling capacity was determined using [22] method. About 100 mg sample was hydrated in a known volume of distilled water (10 mL) in a graduated cylinder. After 18 h, final volume was measured using the Equation (2):

(2) $$\mathrm{SC}\left(\mathrm{mL}/g\right)=\left(\frac{c-b}{a}\right)$$

where SC = swelling capacity, a = sample weight, b = distilled water volume, and c = final volume.

Water/oil absorption capacity was determined using [23] method. About 1 g sample was mixed with 10 mL of distilled water/vegetable oil. Suspension was mixed by vortex and allowed to stand for 30 min. After centrifugation (5000 g/30 min) (6K15 Robot centrifuge, Sigma Inc., Selbyville, DE, USA), supernatant was collected and measured. Water/oil absorption capacity was expressed in mL of water/oil absorbed per g of sample using the Equation (3):

(3) $$\mathrm{WAC}/\mathrm{OAC}\left(\mathrm{mL}/g\right)=\left(\frac{c-b}{a}\right)$$

where WAC = water absorption capacity, OAC = oil absorption capacity, a = sample weight, b = sample volume, c = final volume.

Foaming capacity was determined using [24] method with a slight modification. A total of 1 g flour was added to 50 mL distilled water at 30 ± 2 °C in a graduated cylinder. Suspension was mixed and shaken for 5 min to foam. Foam volume, at 30 s after whipping, was expressed as foam capacity using the Equation (4):

(4) $$\mathrm{FC}\left(\%\right)=\left(\frac{b-a}{a}\right)\times 100$$

where FC = foam capacity, a = foam volume before whipping, b = foam volume after whipping and rest for 30 s.

Foaming stability was determined using [24] method with a slight modification. Final solution volume was recorded 1 h after whipping to determine foam stability using the Equation (5):

(5) $$\mathrm{FS}\left(\%\right)=\left(\frac{b}{a}\right)\times 100$$

where FS = foam stability, a (initial volume) = initial foam volume 30 s after whipping, b (final volume) = foam volume after rest for 1 h.

Least gelation concentration was determined using [25] method. Sample dispersions of 4, 6, 8, 10, 12, and 14% (*w/v*) were prepared in distilled water, adjusted to pH 7.0, and mixed in a Waring Blender at the highest speed for 2 min. About 5 mL each, of dispersions were poured into three test tubes and heated to 100 °C in a water bath for 1 h and cooled to 4 °C in an ice bath. The lowest concentration at which all dispersions in triplicate formed gels that did not collapse or slip from inverted tubes was reported as least gelation concentration.

Gelatinization temperature was determined using [26] method. About 1 g flour was carefully weighed and transferred to 20 mL lidded test tubes. Then, 10 mL water was added to each tube. Then the samples were gently heated in a water bath until a solid gel formed. At the end of gel formation, related temperature was measured and considered as gelatinization temperature.

### 2.4. Technological Parameters

Color measurements were carried out using a calibrated Minolta Color Reader CR 400 (Reader CR-400, Konica Minolta Inc., Tokyo, Japan) [27] based on lightness (L*), redness (a*) and yellowness (b*) values as described by CIE (International Commission on Illumination).

Gluten strength was evaluated using SDS Sedimentation Test according to the Moroccan Standard 08.1.217 [28]. This test is based on reading volume of deposit formed after a series of shaking and swelling of fixed proteins under well-defined conditions, using 6.3 g of ground durum wheat flour, in a solution based on 3% sodium dodecyl sulfate (SDS) and 1.3N lactic acid in the presence of bromophenol blue.

### 2.5. Baking Test

Durum wheat tortilla and composite durum wheat tortilla using 25 and 30% chickpea flours were produced adopting a traditional homemade procedure with the following ingredients: Flour 100 g, salt 1 g, olive oil 20 mL, water 50 mL (variable), and baking powder 0.6 g.

Technological process: Tortilla doughs were prepared based on [29] process with a slight modification; ingredients were mixed with a bread dough mixer (Clatronic KM 3630, Clatronic Inc., New Castle, DE, USA) using a dough hook. Doughs were prepared in triplicate for each test, mixed for 4 min at the 1st speed and for 3 min at the 2nd speed until they became fully developed. Then, doughs were covered with a plastic film and left to rest for 15 min. They were further divided into 50 ± 0.5 g balls, covered with a plastic film, and proofed for 10 min. Finally, tortillas were baked on a griddle for 1 min (variable) on each side, cooled, packed into Ziploc bags, and stored at room temperature.

### 2.6. Nutritional Quality

Moisture, crude ash, and crude proteins were determined following AACC approved methods [12]. Mineral content was determined on dry matter (dm) basis through extraction from samples using dry ashing method [15]. Iron, zinc, copper, and manganese were measured by atomic absorption spectrophotometry [16]. Sodium and potassium contents were estimated using flame photometry [13].

### 2.7. Technological Quality

Amount of water added to flours was measured for each test. Tortilla weights were determined [30], and the diameters and thicknesses were measured before cooking. Cooking time was determined once desired tortilla appearance was obtained. Weights were measured 30 min after removing tortilla from the griddle [30]. Color measurements were carried out using a calibrated Minolta Color Reader CR 400 [27] on the basis of lightness (L*), redness (a*), and yellowness (b*) values as described by CIE (International Commission on Illumination). Tortilla volumes were determined using rape seed displacement method [12]; millet grains were loaded into an empty box with calibrated mark until it reached the marked level and unloaded back. Tortilla was put into the box and the measured millet was loaded back again. Remaining millet grains left outside the box were measured by graduated cylinder and recorded as tortilla volume in cm^3^. Specific volumes were determined by dividing volumes by corresponding weights (cm^3^/g) [31] using the Equation (6):

(6) $$\mathrm{SV}\left(\mathrm{cm}³/g\right)=\left(\frac{a}{b}\right)$$

where SV = specific volume, a = loaf volume, b = loaf weight.

Diameter and thickness were measured after cooking, and color measurement was carried out using a calibrated Minolta Color Reader CR 400 [27].

### 2.8. Effects of Tortilla Making and Baking on Proteins, Minerals, and Technological Parameters

Protein and mineral values in tortillas were compared with those in flours, and their weight, diameter and thickness values were compared with those of doughs.

### 2.9. Effect of Storage Time on Weight, Appearance, Odor and Shelf Stability (Flexibility)

Tortillas were stored under the same conditions at room temperature, wherein weight was measured, and appearance, odor, and flexibility were observed, each day.

### 2.10. Consumer Sensory Appreciation

Tortillas made with 0, 25, and 30% chickpea flours were subjected to sensory assessment by an untrained panel of 50 people accustomed to eating tortillas. Sensory characteristics namely, dark spots, flexibility, puffiness, layering, weight, mouth feel, aroma, and flavor were rated as 1 = excellent, 2 = high, 3 = medium, 4 = poor, and 5 = very poor [32].

### 2.11. Statistical Analysis

Kruskal–Wallis’s test was used to show if there are statistically significant differences between groups, followed by Mann–Whitney test for comparing means; expressed by mean ± SD, using the SPSS software (version 17.0, SPSS Inc., USA). Statistical significance was defined as *p*-value ≤ 0.05. The GraphPad Prism software (version 9.0, GraphPad Prism Inc., San Diego, CA, USA) was used to create graphs. 

## 3. Results and Discussion

### 3.1. Nutritional attributes

Chickpea flour had more ash, proteins, fibers, fat, and energy, and less moisture and carbohydrates than durum wheat flour (Table 1). Adding chickpea flour to durum wheat flour yielded a significant (*p* ≤ 0.05) increase in ash (8.16–26.53%) and proteins (19.88–41.60%) in all combinations, and in fat (22.22%) and energy (0.27%) at 30 and 35% ratios, respectively. In contrast, a significant (*p* ≤ 0.05) decrease in carbohydrates (3.20–7.14%) was observed in all combinations.

Similar trends were obtained for ash, proteins, fibers, fat, carbohydrates, and energy in [33] study. An increase in ash value is likely due to increased amount in minerals [34]. This is required for persons suffering from mineral deficiencies. An increase in protein and fat contents is obviously owing to their higher values in chickpea compared to durum wheat. This is beneficial for growth and development of young people suffering from protein deficiency and provides important fatty acids [35] if values do not exceed daily requirements. An increase in energy value of composite flours is surely due to enhanced protein and fat contents in the blends. A decrease in carbohydrates of various flour combinations is certainly owing to their lower value in chickpea compared to durum wheat. This is good for people suffering from diabetes, overweight, and heart diseases.

**Figure 1:**
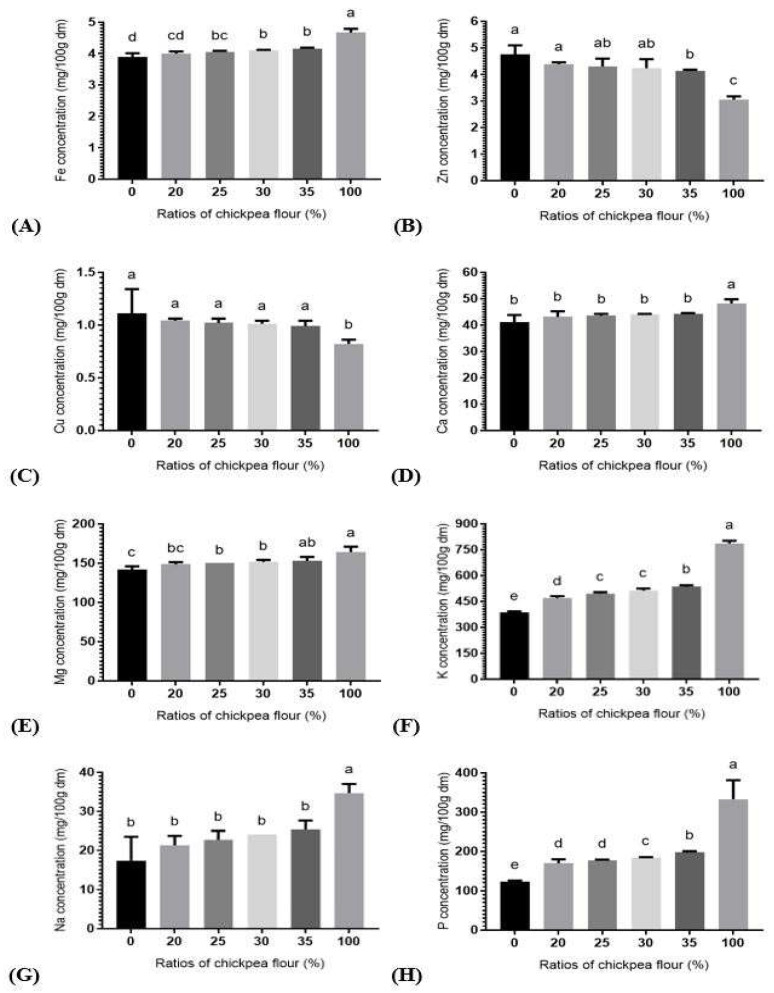
Mineral composition of durum wheat flour, fortified durum wheat flour and chickpea flour; (**A**–**H**) represent Fe, Zn, Cu, Ca, Mg, K, Na, and P concentrations, respectively. Values are mean ± SD (*n* = 3). Means with different superscript letters within the same graph indicate significant statistical differences, *p*-value ≤ 0.05.

Chickpea flour was richer in iron, calcium, magnesium, potassium, sodium, and phosphorus, and lower in zinc and copper than durum wheat flour (Figure 1). Composite flours exhibited significant (*p* ≤ 0.05) increase in both iron (3.84–6.41%) and magnesium (6.03–8.03%) contents at 25% ratio (Figure 1A,E) while potassium (21.80–38.76%) and phosphorus (38.00–61.38%) contents increased in all combinations (Figure 1F,H). In contrary, a significant (*p* ≤ 0.05) decrease was noticed in zinc (13.26%) at 35% ratio (Figure 1B).

Globally, these results are in well agreement with the results of [8]. An increase in minerals of composite flours is attributed to their higher concentration in chickpea than in durum wheat. This justifies the high values obtained for ash and might be useful for individuals with mineral deficiency.

Furthermore, chickpea flour had more phenolic compounds, condensed tannins, total flavonoids, and antiradical-activity than durum wheat flour (Table 2). Composite flours yielded significant (*p* ≤ 0.05) increase in total phenolic compounds (5.88–9.80%), condensed tannins (2.80–4.80%), total flavonoids (66.67–133.33%), and antiradical activity (24.04–50.12%), in all combinations.

These results are in line with the study of [8]. An increase in antioxidants is undoubtedly due to their higher existence in legume coats than in cereals. Phenolic compounds interfere by reducing the risk to have chronic degenerative diseases [36].

### 3.2. Functional Properties

**Figure 2:**
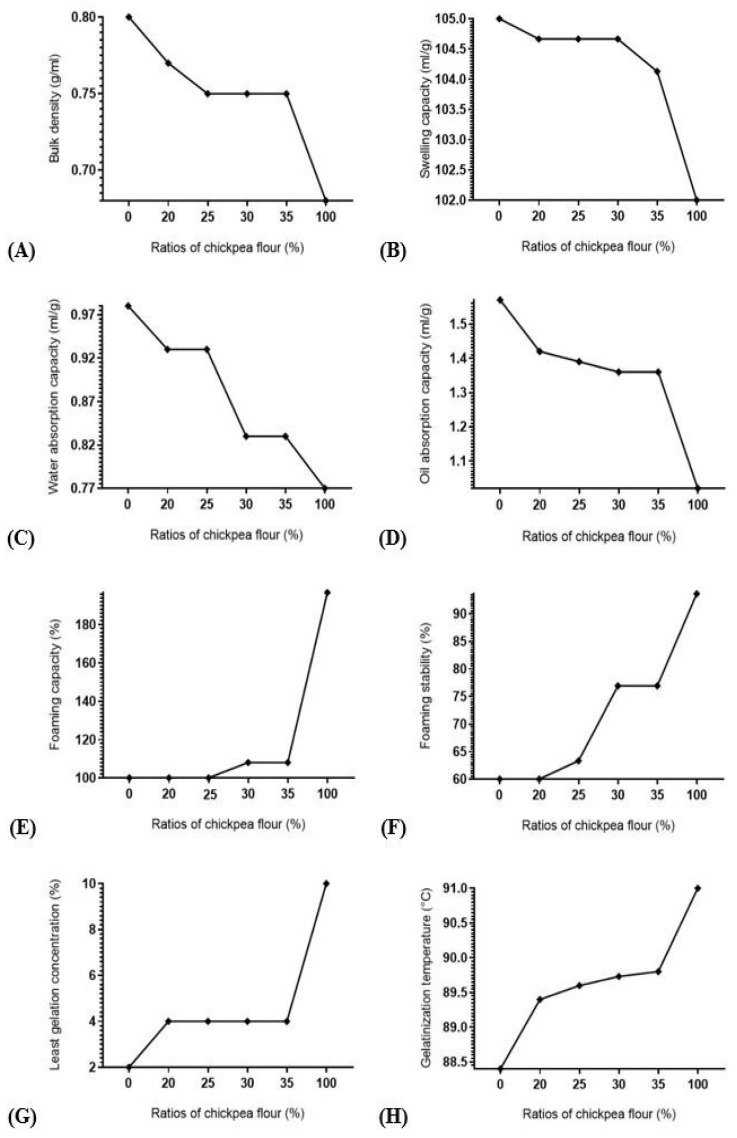
Functional properties values; (**A**): bulk density, (**B**): swelling capacity, (**C**): water absorption capacity, (**D**): oil absorption capacity, (**E**): foaming capacity, (**F**): foaming stability, (**G**): least gelation concentration and (**H**): gelatinization temperature of durum wheat flour, fortified durum wheat flour, and chickpea flour.

The results showed higher foaming capacity, foaming stability, least gelation concentration and gelatinization temperature values in chickpea flour than durum wheat flour, and lower values of bulk density, swelling capacity, and water and oil absorption capacities (Figure 2). Composite flours exhibited significant (*p* ≤ 0.05) increase in foaming capacity (8%) and foaming stability (28.20%) at 30% ratio, least gelation concentration (100%) and gelatinization temperature (1.13–1.59%) in all combinations. In contrary, a significant (*p* ≤ 0.05) decrease was revealed in bulk density (3.75–6.25%) in all combinations, swelling capacity (0.83%) at 35% ratio, water absorption capacity (15.30%) at 30% ratio, and for oil absorption capacity (9.55–13.38%) in all combinations.

These results are in accordance with the results of bulk density, water absorption capacity, oil absorption capacity, foaming properties, least gelation concentration, and gelatinization temperature reported by [37,38,39,40,41,42] studies, respectively. An increase in foaming capacity and foaming stability might be due to the higher protein content in chickpea than in durum wheat. Foaming properties are needed in bread to maintain their texture and structure during processing and throughout the storage [43]. An increase in least gelation concentration could be owing to a change in flour components—such as proteins and carbohydrates—after durum wheat flour substitution. This could be one of the limiting factors in the use of blends in food systems where thickening and gel-forming agents are required [44]. An increase in gelatinization temperature is probably due to addition of other elements such as protein and lipids, obstructing the swelling of granules [42]. This might increase the heat amount needed to reach the final swelling. A decrease in bulk density is probably owing to the difference between spatial arrangement of durum wheat and chickpea particles after being tapped, as it could be due to a reduction in carbohydrate content. The low bulk density would be an advantage in the formulation of complementary foods as a small amount is needed to achieve the required volume of the food product [45]. A decrease in swelling capacity can be attributed to an increase in fat content as it is negatively correlated with swelling power [46] which can be the result of swelling inhibition by amylose [47] as this polysaccharide exists more in legumes starch than in wheat. Lower swelling index indicates lower associative forces [48]. A decrease in water absorption capacity indicates higher hydration ability of durum wheat flour compared to chickpea flour as gluten has the strongest imbibition power compared to other protein sources [49]. The lower the water absorption capacity, the weaker is the flour. A decrease in oil absorption capacity might be due to an increase in fiber content as suggested by many studies [50]. This important functional property improves mouth feel and flavor retention [51] and its decrease is not desirable.

### 3.3. Technological Parameters

Durum wheat flour showed more redness, yellowness, and lightness than chickpea flour (Table 3). Composite flours yielded a significant (*p* ≤ 0.05) increase in redness (3.49%) at 35% ratio and in yellowness (3.93–7.03%) in all combinations.

Similar results were reported in [38] study. The change in the color of flour could be explained by the color differences between durum wheat and chickpea. Yellowness is very preferred by consumers.

**Figure 3:**
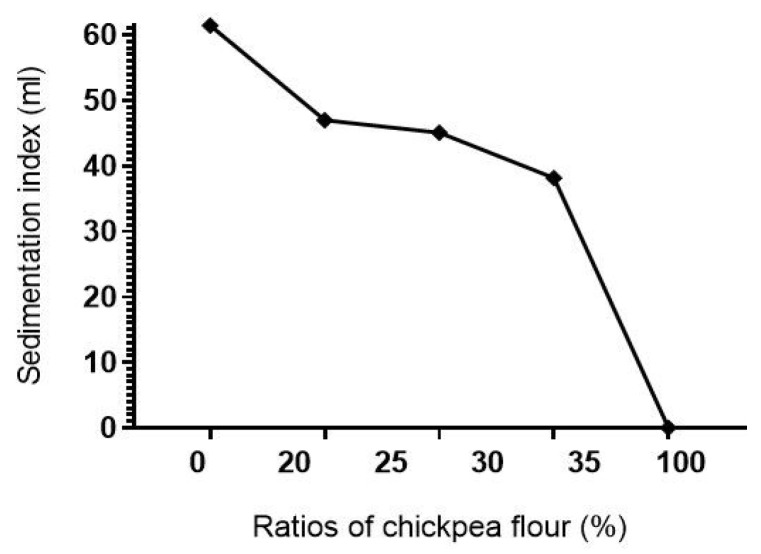
Gluten strength variation of whole durum wheat flour, whole chickpea flour, and their blends.

Sedimentation test values (Figure 3) ranged from “zero” for chickpea flour to 61.40 mL for durum wheat flour. In other words, adding chickpea flour to durum wheat flour reduced significantly (*p* ≤ 0.05) gluten strength.

This decrease in gluten strength is unequivocally due to the replacement of durum wheat gluten by chickpea proteins. Considering quality classes of durum wheat as proposed by [52], 100% durum wheat flour and 20 and 25% chickpea-wheat flour mixtures belong to “excellent” gluten class. While 30 and 35% chickpea-wheat mixtures flours span from “good to very good” gluten class, and 100% chickpea flour belongs to “inadequate” gluten class.

### 3.4. Nutritional Quality

Both, composite tortillas with 25 and 30% chickpea flour showed significantly (*p* ≤ 0.05) more moisture, ash, protein, iron, copper, manganese and potassium, and less zinc than sole durum wheat tortilla. Moreover, tortilla enriched with 30% chickpea flour revealed significantly (*p* ≤ 0.05) more moisture, protein, iron, manganese and potassium, and less zinc than tortilla enriched with 25% chickpea flour (Table 4).

An increase in moisture value is likely due to an increase in air humidity, or to a decrease in cooking time. Moisture of tortilla made from durum wheat enriched with 30% chickpea flour is close to the typical moisture range of wheat tortilla (30–32%) [53]. An increase in ash, protein, iron, copper, manganese, and potassium contents is attributed to their increase in the congruent enriched flours. Except for zinc, these results are useful as healthy diets for persons with protein and mineral deficiencies.

### 3.5. Technological Quality

Durum wheat tortilla had significantly (*p* ≤ 0.05) the high cooking time and low weight values compared to tortilla enriched with chickpea flour. The lowest cooking time and the highest weight values were recorded significantly (*p* ≤ 0.05) for tortilla enriched with 30% chickpea flour, whereas volume and specific volume values were significantly (*p* ≤ 0.05) higher for durum wheat tortilla, and lower for tortilla enriched with 30% chickpea flour. Concerning the parameters of color, enriched tortilla showed significantly (*p* ≤ 0.05) more yellowness (Table 5).

A decrease in added water is maybe due to the higher water absorption capacity of durum wheat flour compared to chickpea flour. An increase in weight value is probably owing to the decrease in cooking time. Obtained weights for tortillas are within the standards. A decrease in cooking time is likely due to the nature of species studied. Enriched tortilla needed less energy than durum wheat tortilla. Volume decrease could be related to a decrease in gluten, which is responsible of the dough’s extensibility [54], it could also be due to starch damage [55]. As specific volume decreases with the decrease in volume, the lower the specific volume the less fluffy and puffy the tortilla is. Variations of color parameters are certainly owing to the nature of studied species and cooking time. Yellowness is very desirable for baked goods.

### 3.6. Effects of Tortillas Making and Baking on Proteins, Minerals, and Technological Parameters

Tortilla processing significantly (*p* ≤ 0.05) increased protein for tortilla enriched with 30% chickpea flour, while minerals except sodium significantly (*p* ≤ 0.05) decreased in all combinations. Baking process significantly (*p* ≤ 0.05) decreased weights and diameters in all combinations.

An increase in protein value is probably caused by the loss of minerals, soluble fibers, and sugars during cooking [56]. An increase in sodium is likely due to the addition of salt during tortilla making. The values did not exceed daily needs (0.12–1.5 g/day) [57]. A decrease in weight and diameter might be due to water evaporation (Table 6). The values did not exceed the standards.

### 3.7. Storage Time Effect on Weight, Appearance, Odor and Shelf Stability

**Figure 4:**
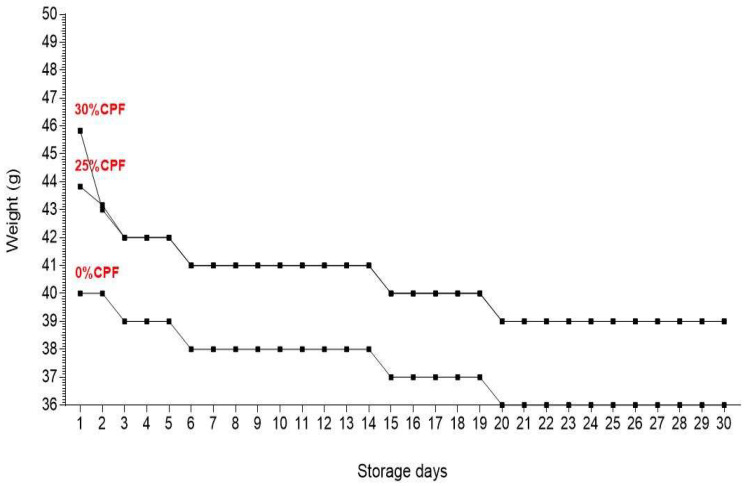
Weight variation of durum wheat tortilla and fortified durum wheat tortillas during storage.

Results presented in Figure 4 and Table 7 showed that the weights of enriched tortilla decreased by 0.66 g for 25% ratio and 2.83 g for 30% ratio on the 2nd day, and by 1 g for durum wheat tortilla, 1.17 g for 25% ratio and 1 g for 30% ratio on the 3rd day. Then, weights stabilized (3rd–5th day); decreased by 1 g and stabilized (6–14th day); decreased by 1 g and stabilized (15–19th day); decreased by 1 g, and stabilized finally during 20–30th day. Regarding appearance and odor, tortilla enriched with 30% chickpea flour showed light green and black dots on the 15th day which became darker day by day occupying more area, with a change in odor from the 6th day and a production of a bad odor from the 26th day. The other ratios changed in odor from the 26th day. Regarding flexibility, tortillas were foldable on the 1st day, then hardness increased over time.

The weights obtained on the 2nd day are likely because gluten has the strongest imbibition power compared to protein from other sources [49]. The decrease observed in the following days could be because of storage conditions on the moisture content of grain. Tortillas weights remained within the norms. Early change in appearance and odor for enriched tortilla with 30% chickpea flour is certainly due to higher moisture content at 30% ratio as compared to the other ratios, which presents a suitable area for mold development. A decrease in flexibility is attributed to moisture loss. Softness is required for tortillas.

### 3.8. Sensory Appreciation

Based on the previous results, tortillas were prepared with enriched chickpea flours of 25 and 30% ratios. Assessment of sensorial quality traits (Table 8) revealed that durum wheat tortilla was rated “high to excellent” for weight; “medium to high” for dark spots, flexibility, puffiness, mouth feel, aroma, and flavor; and “poor to medium” for layering. Tortilla enriched with 25% chickpea flour was rated “high to excellent” for dark spots and weight; “medium to high” for flexibility, mouth feel, aroma, and flavor; and “poor to medium” for puffiness and layering. Tortilla enriched with 30% chickpea flour was rated “high to excellent” for weight and flavor; “medium to high” for dark spots, mouth feel and aroma; and “poor to medium” for flexibility, puffiness, and layering. Briefly, durum wheat tortilla was the most preferred for puffiness and weight, while tortilla enriched with 25% chickpea flour was the most preferred for dark spots, flexibility, layering, mouth feel, and aroma; and tortilla enriched with 30% chickpea flour was the most preferred for flavor. A third of consumers commented that tortilla enriched with 30% chickpea flour melts in mouth being fragile. Most of consumers found enriched tortillas with 25 and 30% chickpea flour more delicious than durum wheat tortilla, and only one consumer felt the taste of ″Karantika″, which is a famous meal made with chickpea flour in East-Morocco.

## 4. Conclusions

This study aimed to produce enriched tortilla from durum wheat-chickpea flours. Based on the quality analysis of durum wheat-chickpea composite flours using different combinations, 25 and 30% ratios of chickpea flour were chosen for making enriched tortillas. Nutritionally, many improvements were obtained especially higher ash, protein, iron, copper, manganese, and potassium, being beneficial for malnourished people. Technologically, 30% ratio yielded better results for yellowness and cooking time, and weights were not negatively influenced. In addition, tortillas making and cooking improved protein content, while weights and diameters were not negatively influenced. During storage, no negative impact on tortillas weights was observed, but their decrease led to a decrease in flexibility, and sanitary quality was lost early for 30% chickpea flour. Sensorially, the choice of enriched tortillas differs with consumers based on the evaluated sensorial characteristics. Overall, durum wheat-chickpea-enriched tortilla is feasible, providing an innovative cereal product with added nutritional value, while keeping or even improving many parameters for other aspects of quality. So, the amalgamation of legumes-based products into our daily diet is recommended, and further research in novel durum wheat-pulses-based products with high nutritive value should be strengthened.

## Figures and Tables

**Table 1:** Physicochemical composition of durum wheat flour, fortified durum wheat flour, and chickpea flour.

Combinations	Parameters						
	MS (%, dm)	CA (%, dm)	CP (%, dm)	CFb (%, dm)	CF (%, dm)	TC (%, dm)	EV (kcal/100 g, dm)
100%WF	9.55 ± 0.41 ^abc^	1.96 ± 0.08 ^f^	11.37 ± 0.15 ^f^	3.47 ± 0.28 ^bc^	1.80 ± 0.20 ^d^	75.32 ± 0.82 ^a^	362.97 ± 1.01 ^c^
20%CPF:80%WF	9.40 ± 0.02 ^a^	2.12 ± 0.03 ^e^	13.63 ± 0.06 ^e^	3.54 ± 0.02 ^c^	1.93 ± 0.12 ^d^	72.91 ± 0.20 ^b^	363.57 ± 0.45 ^bc^
25%CPF:75%WF	9.36 ± 0.01 ^b^	2.24 ± 0.05 ^d^	14.43 ± 0.15 ^d^	3.56 ± 0.01 ^c^	2.03 ± 0.06 ^cd^	71.92 ± 0.25 ^c^	363.72 ± 0.19 ^bc^
30%CPF:70%WF	9.32 ± 0.02 ^c^	2.37 ± 0.04 ^c^	15.27 ± 0.15 ^c^	3.59 ± 0.02 ^b^	2.13 ± 0.06 ^bc^	70.94 ± 0.18 ^d^	363.91 ± 0.36 ^bc^
35%CPF:65%WF	9.29 ± 0.03 ^c^	2.48 ± 0.03 ^b^	16.10 ± 0.10 ^b^	3.62 ± 0.02 ^b^	2.20 ± 0.00 ^b^	69.94 ± 0.16 ^e^	363.95 ± 0.23 ^b^
100%CPF	9.07 ± 0.11 ^d^	3.15 ± 0.09 ^a^	23.17 ± 0.15 ^a^	4.03 ± 0.06 ^a^	2.79 ± 0.20 ^a^	61.83 ± 0.32 ^f^	365.12 ± 0.97 ^a^

WF: durum wheat flour; CPF: chickpea flour; MS: moisture; CA: crude ash; CP: crude proteins; CFb: crude fibers; CF: crude fat; TC: total carbohydrates; EV: energy value. Values are mean ± SD (*n* = 3). Means with different superscript letters within the same column indicate significant statistical differences, *p*-value ≤ 0.05.

**Table 2:** Total phenolic compounds, condensed tannins, total flavonoids, and antiradical-activity of durum wheat flour, fortified durum wheat flour, and chickpea flour.

Combinations	Parameters			
	TPC (mg GAE/g dm)	CT (mg CE/g dm)	TF (mg QE/g dm)	ARA (% dm)
100%WF	0.51 ± 0.01 ^d^	2.50 ± 0.03 ^d^	0.03 ± 0.00 ^e^	28.29 ± 0.51 ^f^
20%CPF:80%WF	0.54 ± 0.01 ^c^	2.57 ± 0.03 ^c^	0.05 ± 0.00 ^d^	35.09 ± 1.70 ^e^
25%CPF:75%WF	0.55 ± 0.00 ^c^	2.58 ± 0.00 ^c^	0.06 ± 0.00 ^c^	37.16 ± 0.17 ^d^
30%CPF:70%WF	0.55 ± 0.01 ^bc^	2.59 ± 0.01 ^bc^	0.06 ± 0.00 ^b^	40.02 ± 1.29 ^c^
35%CPF:65%WF	0.56 ± 0.01 ^b^	2.62 ± 0.02 ^b^	0.07 ± 0.00 ^b^	42.47 ± 0.71 ^b^
100%CPF	0.66 ± 0.05 ^a^	2.74 ± 0.11 ^a^	0.14 ± 0.00 ^a^	58.03 ± 0.38 ^a^

WF: durum wheat flour; CPF: chickpea flour; TPC: total phenolic compounds; CT: condensed tannins; TF: total flavonoids; ARA: antiradical-activity. Solvent used to prepare extracts: acetone/water. Values are mean ± SD (*n* = 3). Means with different superscript letters within the same column indicate significant statistical differences, *p*-value ≤ 0.05.

**Table 3:** Color parameters measurement of durum wheat flour, fortified durum flour, and chickpea flour.

Combinations	Parameters		
	a	b	L
100%WF	−1.43 ± 0.02 ^c^	16.50 ± 0.02 ^f^	68.78 ± 0.36 ^bcd^
20%CPF:80%WF	−1.41 ± 0.01 ^c^	17.15 ± 0.02 ^e^	68.86 ± 0.02 ^d^
25%CPF:75%WF	−1.39 ± 0.02 ^bc^	17.32 ± 0.02 ^d^	68.88 ± 0.01 ^cd^
30%CPF:70%WF	−1.39 ± 0.02 ^bc^	17.49 ± 0.01 ^c^	68.90 ± 0.01 ^c^
35%CPF:65%WF	−1.38 ± 0.00 ^b^	17.66 ± 0.01 ^b^	68.93 ± 0.01 ^b^
100%CPF	−1.25 ± 0.00 ^a^	19.97 ± 0.06 ^a^	69.30 ± 0.01 ^a^

WF: durum wheat flour; CPF: chickpea flour; a: redness; b: yellowness; L: lightness. Values are mean ± SD (*n* = 3). Means with different superscript letters within the same column indicate significant statistical differences, *p*-value ≤ 0.05.

**Table 4:** Nutritional composition of durum wheat tortilla and fortified durum wheat tortillas.

Parameters	Combinations		
	100%WF	25%CPF:75%WF	30%CPF:70%WF
MS (%, dm)	17.74 ± 0.30 ^c^	22.08 ± 2.19 ^b^	25.73 ± 0.64 ^a^
CA (%, dm)	1.97 ± 0.01 ^b^	2.29 ± 0.50 ^a^	2.55 ± 0.43 ^a^
CP (%, dm)	11.50 ± 0.00 ^c^	14.50 ± 0.00 ^b^	15.70 ± 0.00 ^a^
Fe (mg 100 g^−1^ dm)	3.35 ± 0.01 ^c^	3.61 ± 0.01 ^b^	3.80 ± 0.00 ^a^
Zn (mg 100 g^−1^ dm)	4.04 ± 0.01 ^a^	3.65 ± 0.01 ^b^	3.62 ± 0.01 ^c^
Cu (mg 100 g^−1^ dm)	0.53 ± 0.00 ^a^	0.62 ± 0.00 ^b^	0.62 ± 0.00 ^b^
Mn (mg 100 g^−1^ dm)	1.00 ± 0.01 ^c^	1.32 ± 0.01 ^b^	1.40 ± 0.00 ^a^
K (mg 100 g^−1^ dm)	282.67 ± 4.62 ^c^	413.87 ± 0.46 ^b^	437.33 ± 4.62 ^a^
Na (mg 100 g^−1^ dm)	986.67 ± 11.54 ^a^	993.33 ± 11.54 ^a^	996.00 ± 6.92 ^a^

WF: durum wheat flour; CPF: chickpea flour; MS: moisture; CA: crude ash; CP: crude proteins. Values are mean ± SD (*n* = 3). Means with different superscript letters within the same line indicate significant statistical differences, *p*-value ≤ 0.05.

**Table 5:** Technological quality of durum wheat tortilla and fortified durum wheat tortillas with chickpea flour.

Parameters	Combinations		
	100%WF	25%CPF:75%WF	30%CPF:70%WF
Added water (mL) to the dough	50.00	46.00	44.00
Cooking time (min) each tortilla side	1.5 ± 0.00 ^a^	1.2 ± 0.00 ^b^	1.06 ± 0.00 ^c^
Weight (g)	40.00 ± 0.00 ^c^	44.00 ± 0.00 ^b^	46.00 ± 0.00 ^a^
Diameter (cm)	15.00 ± 0.00 ^a^	15.00 ± 0.00 ^a^	15.00 ± 0.00 ^a^
Thickness (mm)	2.00 ± 0.00 ^a^	2.00 ± 0.00 ^a^	2.00 ± 0.00 ^a^
Volume (cm^3^)	60.33 ± 0.58 ^a^	52.00 ± 0.00 ^b^	49.67 ± 0.58 ^c^
Specific volume (cm^3^/g)	1.51 ± 0.01 ^a^	1.18 ± 0.00 ^b^	1.08 ± 0.01 ^c^
a	2.41 ± 0.05 ^c^	2.63 ± 0.10 ^b^	3.61 ± 0.24 ^a^
b	32.28 ± 0.54 ^b^	34.34 ± 0.43 ^b^	35.01 ± 0.33 ^a^
L	65.41 ± 0.59 ^a^	60.10 ± 1.05 ^b^	58.51 ± 0.91 ^b^

WF: durum wheat flour; CPF: chickpea flour; a: redness; b: yellowness; L: lightness. Values are mean ± SD (*n* = 3). Means with different superscript letters within the same line indicate significant statistical differences, *p*-value ≤ 0.05.

**Table 6:** Proteins and minerals in flours and tortillas, and technological parameters of doughs and tortillas.

Parameters		Combinations					
		100%WF		25%CPF:75%WF		30%CPF:70%WF	
Added water (mL)		50.00		46.00		44.00	
Cooking time (min) each side		1.5 ± 0.00 ^a^		1.2 ± 0.00 ^b^		1.06 ± 0.00 ^c^	
Parameters/Elements		Flour	Tortilla	Flour	Tortilla	Flour	Tortilla
Proteins (%, dm)		11.37 ± 0.15 ^a^	11.50 ± 0.00 ^a^	14.43 ± 0.15 ^a^	14.50 ± 0.00 ^a^	15.27 ± 0.15 ^b^	15.70 ± 0.00 ^a^
Minerals (mg 100 g^−1^ dm)	Fe	3.90 ± 0.11 ^a^	3.35 ± 0.01 ^b^	4.05 ± 0.04 ^a^	3.61 ± 0.01 ^b^	4.11 ± 0.01 ^a^	3.80 ± 0.00 ^b^
	Zn	4.75 ± 0.34 ^a^	4.04 ± 0.01 ^b^	4.30 ± 0.29 ^a^	3.65 ± 0.01 ^b^	4.23 ± 0.34 ^a^	3.62 ± 0.01 ^b^
	Cu	1.11 ± 0.23 ^a^	0.53 ± 0.00 ^b^	1.02 ± 0.04 ^a^	0.62 ± 0.00 ^b^	1.01 ± 0.03 ^a^	0.62 ± 0.00 ^b^
	K	385.33 ± 6.11 ^a^	282.67 ± 4.62 ^b^	494.67 ± 8.33 ^a^	413.87 ± 0.46 ^b^	512.00 ± 12.00 ^a^	437.33 ± 4.62 ^b^
	Na	17.33 ± 6.11 ^b^	986.67 ± 11.54 ^a^	22.67 ± 2.31 ^b^	993.33 ± 11.54 ^a^	24.00 ± 0.00 ^b^	996.00 ± 6.92 ^a^
Parameters/Elements		*Dough*	*Tortilla*	*Dough*	*Tortilla*	*Dough*	*Tortilla*
Weight (g)		50.00 ± 0.00 ^a^	40.00 ± 0.00 ^b^	50.00 ± 0.00 ^a^	44.00 ± 0.00 ^b^	50.00 ± 0.00 ^a^	46.00 ± 0.00 ^b^
Diameter (cm)		16.00 ± 0.00 ^a^	15.00 ± 0.00 ^b^	16.00 ± 0.00 ^a^	15.00 ± 0.00 ^b^	16.00 ± 0.00 ^a^	15.00 ± 0.00 ^b^
Thickness (mm)		2.00 ± 0.00 ^a^	2.00 ± 0.00 ^a^	2.00 ± 0.00 ^a^	2.00 ± 0.00 ^a^	2.00 ± 0.00 ^a^	2.00 ± 0.00 ^a^

WF: durum wheat flour; CPF: chickpea flour. Values are mean ± SD (*n* = 3). Means with different superscript letters for the same parameter and ratio indicate significant statistical differences, *p*-value ≤ 0.05.

**Table 7:** Appearance, odor, and flexibility variations of durum wheat tortilla and fortified durum wheat tortillas during storage. CPF: chickpea flour. Values are mean ± SD (*n* = 3).

Storage Days/ Parameters/ Combinations	Appearance			Odor			Flexibility		
	100%WF	25%CPF	30%CPF	100%WF	25%CPF	30%CPF	100%WF	25%CPF	30%CPF
1st	NL	NL	NL	NL	NL	NL	FLD	FLD	FLD
2nd–5th	NL	NL	NL	NL	NL	NL	N.FLD	N.FLD	N.FLD
6th–14th	NL	NL	NL	NL	NL	O.ch	N.FLD	N.FLD	N.FLD
15th–25th	NL	NL	C.Ch	NL	NL	O.Ch	N.FLD	N.FLD	N.FLD
26th–30th	NL	NL	C.Ch	O.Ch	O.Ch	O.Ch	N.FLD	N.FLD	N.FLD

WF: durum wheat flour; CPF: chickpea flour; NL: normal; C.Ch: color change; O.Ch: odor change; FLD: foldable; N.FLD: not foldable.

**Table 8:** Sensorial quality traits of durum wheat tortilla and fortified durum wheat tortillas.

Parameters	Combinations		
	100%WF	25%CPF:75%WF	30%CPF:70%WF
Dark spots	2.86 ± 1.42	1.66 ± 0.82	2.08 ± 1.08
Flexibility	2.64 ± 1.28	2.30 ± 0.93	3.20 ± 1.38
Puffiness	2.62 ± 1.14	3.38 ± 1.44	3.86 ± 1.17
Layering	3.98 ± 0.14	3.94 ± 0.23	4.00 ± 0.00
Weight	1.16 ± 0.61	1.38 ± 0.72	1.36 ± 0.72
Mouth feel	2.50 ± 1.11	2.14 ± 1.19	2.46 ± 1.28
Aroma	2.26 ± 1.04	2.06 ± 1.07	2.56 ± 1.45
Flavor	2.24 ± 1.11	2.50 ± 1.31	1.40 ± 0.49

WF: durum wheat flour; CPF: chickpea flour. Values are mean ± SD (*n* = 3).

## Data Availability

Data is contained within the article.

## References

[1] Taghouti M., Nsarellah N., Rhrib K., Benbrahim N., Amallah L., Rochdi A. (2017). Evolution from durum wheat landraces to recent improved varieties in Morocco in terms of productivity increase to the detriment of grain quality. Revue Marocaine des Sciences Agronomiques et Vétérinaires.

[2] Aboussaleh Y., Farsi M., El Hioui M., Ahami A. (2009). Transition nutritionnelle au Maroc: Coexistence de l’anémie et de l’obésité chez les femmes au Nord-Ouest marocain. Antropo.

[3] Allali F. (2017). Evolution des pratiques alimentaires au Maroc nutrition transition in Morocco. Int. J. Med. Surg..

[4] Aboussaleh Y., Sbaibi R., El Hioui M., Ahami A. (2011). La carence en fer et le développement cognitif. Antropo.

[5] Achouri I., Aboussaleh Y., Sbaibi R., El Hioui M., Ahami A. (2015). Prevalence of iron deficiency anemia and associated factors among urban school children in Kenitra, northwest of Morocco. Pak. J. Biol. Sci..

[6] Chadare F.J., Idohou R., Nago E., Affonfere M., Agossadou J., Fassinou T.K., Kénou C., Honfo S., Azokpota P., Linnemann A.R. (2019). Conventional and food-to-food fortification: An appraisal of past practices and lessons learned. Food Sci. Nutr..

[7] Benali A., En-nahli Y., Noutfia Y., Elbaouchi A., Kabbour M.R., Gaboun F., El Maadoudi E.H., Benbrahim N., Taghouti M., Ouhssine M. (2021). Nutritional and Technological Optimization of Wheat-Chickpea-Milk Powder Composite Flour and Its Impact on Rheological and Sensorial Properties of Leavened Flat Bread. Foods.

[8] Benayad A., Taghouti M., Benali A., Aboussaleh Y., Benbrahim N. (2020). Nutritional and technological assessment of durum wheat-faba bean enriched flours, and sensory quality of developed composite bread. Saudi J. Biol. Sci..

[9] Bouhlal O., Taghouti M., Benbrahim N., Benali A., Visioni A., Benba J. (2019). Wheat-lentil fortified flours: Health benefits, physicochemical, nutritional and technological properties. J. Mater. Environ. Sci..

[10] Maphosa Y., Jideani V.A., María C.H. (2017). The role of legumes in human nutrition. Book of Functional Food-Improve Health through Adequate Food.

[11] Piga A., Conte P., Fois S., Catzeddu P., Del Caro A., Sanguinetti A.M., Fadda C. (2021). Technological, nutritional and sensory properties of an innovative gluten-free double-layered flat bread enriched with amaranth flour. Foods.

[12] AACC (American Association of Cereal Chemists) (2000). Approved Methods of the AACC.

[13] AOAC (Association of Official Analytical Chemists) (2005). Official Methods of Analysis of Association of Official Analytical Chemists.

[14] Osborne D.R., Voogt P., Osborne D.R., Voogt P. (1978). Calculation of caloric value. The Analysis of Nutrients in Foods.

[15] Chapman H.D., Pratt F.P., Chapman H.D., Pratt F.P. (1982). Determination of minerals by titration method. Methods of Analysis for Soils, Plants and Water.

[16] AOAC (1990). Official Methods of Analysis.

[17] Khalil I.A., Manan F. (1990). Chemistry-One (Bio-Analytical Chemistry).

[18] Singleton V.L., Orthofer R., Lamuela-Raventos R.M. (1999). Analysis of total phenols and other oxidation substrates and antioxidants by means of Folin-Ciocalteu reagent. Meth. Enzymol..

[19] Julkunen-titto R. (1985). Phenolic constituents in the leaves of northern willows: Methods for the analysis of certain phenolics. J. Agric. Food Chem..

[20] Mansouri A., Embarek G., Kokkalou E., Kefalas P. (2005). Phenolic profile and antioxidant activity of the Algerian ripe date palm fruit (*Phoenix dactylifera*). Food Chem..

[21] Onwuka G.I. (2005). Food Analysis and Instrumentation: Theory and Practice.

[22] Robertson J.A., DE Monredon F.D., Dysseler P., Guillon F., Amado R., Thibault J.F. (2000). Hydration Properties of Dietary Fibre and Resistant Starch: A European collaborative study. LWT—Food Sci. Technol..

[23] Beuchat L.R. (1977). Functional and electrophoretic characteristics of succinylated peanut flour protein. J. Agric. Food Chem..

[24] Narayana K., Narsinga, Rao M.S. (1982). Functional properties of war and heat processed winged bean (*Psophocarpus tetragonolobus*) flour. J. Food Sci..

[25] Mugendi J.B.W., Njagi E.N.M., Kuria E.N., Mwasaru M.A., Mureithi J.G., Apostolides Z. (2010). Nutritional quality and physicochemical properties of Mucuna bean (*Mucuna pruriens L.*) protein isolates. Int. Food Res. J..

[26] Shinde B.G. (2001). Isolation and Characterization of Starch Horse Gram. Unpublished Master’s Thesis.

[27] (2008). Colorimetry, Part 4: Color Space L * a * b * CIE 1976 (Classification Index: T36-007-4PR).

[28] AACC (1984). Approved Methods of the AACC.

[29] Hernández-López I., Benavente Valdés J.R., Castellari M., Aguiló-Aguayo I., Morillas-España A., Sánchez-Zurano A., Acién-Fernández F.G., Lafarga T. (2021). Utilisation of the marine microalgae *Nannochloropsis* sp. and *Tetraselmis* sp. as innovative ingredients in the formulation of wheat tortillas. Algal Res..

[30] Makinde F.M., Akinoso R. (2014). Physical, nutritional and sensory qualities of bread samples made with wheat and black sesame (*Sesamum indicum Linn*) flours. Int. Food Res. J..

[31] Araki E., Ikda T.M., Ashida K., Takata K., Yanaka M., Iida S. (2009). Effects of rice flour properties on specific loaf volume of one-loaf bread made from rice flour with wheat vital gluten. Food Sci. Technol. Res..

[32] Bello A.B., Senna-Saldivar S.O., Waniska R.D., Rooney L.W. (1991). Methods to prepare and evaluate wheat tortillas. Cereal Foods World..

[33] Bayomy H., Alamri E. (2022). Technological and nutritional properties of instant noodles enriched with chickpea or lentil flour. J. King Saud Univ. Sci..

[34] Liu K. (2019). Effects of sample size, dry ashing temperature and duration on determination of ash content in algae and other biomass. Algal Res..

[35] Madurapperumage A., Tang L., Thavarajah P., Bridges W., Shipe E., Vandemark G., Thavarajah D. (2021). Chickpea (*Cicer arietinum L.*) as a source of essential fatty acids—A biofortification approach. Front. Plant Sci..

[36] Gutierrez-Grijalva E.P., Ambriz-Pere D.L., Leyva-Lopez N., Castillo-Lopez R.I., Heiedia J.B. (2016). Review: Dietary phenolic compounds, health benefits and bioaccessibility. Arch. Latinoam. Nutr..

[37] Boucheham N., Galet L., Patry S., Zidoune M.N. (2019). Physicochemical and hydration properties of different cereal and legume gluten-free powders. Food Sci. Nutr..

[38] Yıldırım A., Karaboğa Z.Y. The effects of corn and chickpea flours on the quality of mardin peksimet. Proceedings of the International Conference on Food, Agriculture and Animal Husbandry.

[39] Diallo S., Doudjo S., Youssouf K.K., Emmanuel A.N., Benjamin Y.K., Dago G. (2015). Fortification et substitution de la farine de blé par la farine de Voandzou (*Vigna subterranea L. verdc*) dans la production des produits de boulangerie. Int. J. Innov. Sci. Res..

[40] Stone A.K., Nosworthy M.G., Chiremba C., House J.D., Nickerson M.T. (2019). A comparative study of the functionality and protein quality of a variety of legume and cereal flours. Cereal. Chem..

[41] Orisa C.A., Udofia S.U. (2020). Functional and pasting properties of composite flours from *Triticum durum*, *Digitaria exilis*, *Vigna unguiculata* and *Moringa oleifera Powder*. Asian Food Sci. J..

[42] Falade K.O., Okafor C.A. (2015). Physical, functional, and pasting properties of flours from corms of two Cocoyam (*Colocasiaesculenta and Xanthosoma sagittifolium*) cultivars. Food Sci. Technol..

[43] Nawaz H., Shad M.A., Mehmood R., Rehman T., Munir H. (2015). Comparative evaluation of functional properties of some commonly used cereal and legume flours and their blends. Int. J. Food Allied Sci..

[44] Ocheme O.B., Oloyede O.O., Mahmud A.H. (2010). Production and evaluation of bread usingblends of wheat flour and fermented plantain flour. Niger. Food J..

[45] Suresh C. (2013). Assessment of functional properties of different flours. Afr. J. Agric. Res..

[46] Culetu A., Susman I.E., Duta D.E., Belc N. (2021). Nutritional and functional properties of gluten-free flours. Appl. Sci..

[47] Chaiyakul S., Sukkasem D., Natthapanpaisith P. (2016). Effect of flour concentration and retrogradation treatment on physical properties of instant sinlek brown rice. Int. J. Food Eng..

[48] Buckman E.S., Oduro I., Plahar W.A., Tortoe C. (2017). Determination of the chemical and functional properties of yam bean (*Pachyrhizus erosus (L.) Urban*) flour for food systems. Food Sci. Nutr..

[49] Yousseff S.A.M., Salem A., Abdel-Rahman A.H.Y. (1976). Supplementation of bread with soybean and chickpea flours. Int. J. Food Sci. Technol..

[50] Brannan R.G., Mah E., Schott M., Yuan S., Casher K.L., Myers A., Herrick C. (2014). Influence of ingredients that reduce oil absorption during immersion frying of battered and breaded foods. Eur. J. Lipid Sci. Technol..

[51] Iwe M.O., Onyeukwu U., Agiriga A.N. (2016). Proximate, functional and pasting properties of FARO 44 rice, African yam bean and brown cowpea seeds composite flour. Cogent Food Agric..

[52] Cubadda R.E., Carcea M., Marconi E., Trivisonno M.C. (2007). Influence of protein content on durum wheat gluten strength determined by the SDS Sedimentation Test and by other methods. Cereal Food World..

[53] Brooker D. (2010). Tortilla Tips: Moisture Content of Tortillas. https://www.tortillanews.com/2010/04/tortilla-tips-moisture-content-of-tortilla.

[54] Alviola J.N., Waniska R.D., Rooney L.W. (2008). Role of gluten in flour tortilla staling. Cereal Chem..

[55] Jukić M., Komlenić D.K., Mastanjević K., Mastanjević K., Lučan M., Popovici C., Nakov G., Lukinac J. (2019). Influence of damaged starch on the quality parameters of wheat doughand bread. Food Technol..

[56] Baik B.K., Han I.H. (2012). Cooking, Roasting, and Fermentation of Chickpeas, Lentils, Peas, and Soybeans for Fortification of Leavened Bread. Cereal Chem..

[57] IOM (Institute of Medicine) (2005). EBook of Dietary Reference intakes for Water, Potassium, Sodium, Chloride, and Sulfate.

